# Navigating unique challenges and advancing equitable care for children with ADHD in Africa: a review

**DOI:** 10.1097/MS9.0000000000001179

**Published:** 2023-08-14

**Authors:** Gbolahan Olatunji, Olamide Faturoti, Babafemi Jaiyeoba, Ayodele V. Toluwabori, Temiloluwa Adefusi, Peter Olaniyi, Nicholas Aderinto, Muili O. Abdulbasit

**Affiliations:** a Department of Medicine and Surgery, University of Ilorin, Ilorin; b Bowen University Teaching Hospital; c Department of Medicine and Surgery, Ladoke Akintola University of Technology, Ogbomoso, Nigeria

**Keywords:** Africa, attention-deficit/hyperactivity disorder, neurology

## Abstract

Attention-deficit/hyperactivity disorder (ADHD) is a neurodevelopmental condition that presents distinct challenges for African children due to a complex interplay of social, economic and cultural factors. This review examines the various obstacles faced by children with ADHD in Africa, focusing on the lack of awareness and stigma surrounding the disorder, limited access to mental health services, educational system constraints, economic limitations and cultural beliefs and practices. The review underscores the significance of public awareness campaigns and educational initiatives to dispel myths and misconceptions surrounding ADHD. These efforts should target the general public, educators, healthcare professionals, religious leaders and traditional healers. Crucial to addressing this issue is strengthening mental health services, especially in rural and underserved areas, through increased funding and the availability of trained mental health professionals specialised in ADHD diagnosis and treatment. Integrating mental health services into primary healthcare systems is proposed to enhance accessibility for children with ADHD. By training primary care providers to recognise ADHD symptoms and provide initial support and referrals, early identification and intervention can be facilitated. Addressing the affordability and accessibility of ADHD treatment is a priority, with policy recommendations including subsidising medications and therapies and providing financial assistance to families in need. A call for collaborative efforts between governments, nongovernmental organisations and pharmaceutical companies is advocated to ensure that ADHD treatment is affordable and widely available.

## Introduction

Highlights

- Cultural beliefs, stigma and limited access to mental health services often result in the underdiagnosis or misdiagnosis of children with attention-deficit/hyperactivity disorder (ADHD).
- Understanding these disparities is crucial for developing targeted interventions to ensure that children with ADHD receive appropriate care and support.
- Potential solutions, such as telemedicine, community-based care models and partnerships with nongovernmental organisations can help bridge the gap and ensure more equitable access to care for children with ADHD across the continent.

Attention-deficit/hyperactivity disorder (ADHD) significantly impacts the lives of many children and adolescents worldwide^1^. It is characterised by impairing inattention, disorganisation and/or hyperactivity-impulsivity, as defined in the DSM-5^2^. ADHD’s features include challenges in attentional endurance, motor hyperactivity and heightened impulsivity, while its aetiology is complex, stemming from multiple genetic and environmental factors^2^.

Recent recognition of the importance of understanding and addressing ADHD in Africa has led to studies exploring its prevalence and impact on the continent. Globally, ADHD affects around 6% of children and adolescents and 3% of adults^3^. However, specific African populations exhibit varying rates, ranging from 1.5% in the general populace to 100% in special populations of children with apparent organic brain injury^3^. Despite these ongoing investigations, research on ADHD in Africa remains limited, and unique challenges persist in understanding its prevalence, risk factors and cultural implications. Understanding ADHD in Africa is vital to providing equitable care for affected children, as untreated ADHD can lead to various complications affecting academic performance, social skills, self-esteem, and overall well-being.

This review explores the current knowledge regarding ADHD in Africa, focusing on its prevalence, risk factors, comorbidities and cultural aspects influencing its manifestation and management. By meticulously examining the available literature, including studies from diverse African countries, we aim to comprehensively understand ADHD within the African context and identify potential avenues for improving equitable and effective care for affected children. Additionally, this review will highlight the importance of cultural factors in shaping the presentation and treatment of ADHD in African communities.

Synthesising existing research on ADHD in Africa will contribute to collective efforts to enhance mental healthcare for children with ADHD across the continent. By identifying gaps in knowledge and understanding, we hope to nurture future research endeavours that can lead to tailored interventions, promoting positive outcomes for children with ADHD and enhancing their overall well-being in African communities.

## Methodology

This study is a narrative review. A comprehensive literature search was conducted to achieve this objective, encompassing electronic databases such as PubMed, PsycINFO and Google Scholar (Table 1). The search used a combination of keywords, including ʻADHDʼ, ʻattention-deficit/hyperactivity disorderʼ, ʻprevalenceʼ, ʻAfricaʼ, ʻsub-Saharan Africaʼ, ʻNorth Africaʼ, ʻchildrenʼ, and ʻadolescentsʼ. The inclusion criteria for studies in this review were as follows:

1. Focus on ADHD prevalence among children and adolescents in African countries.
2. Inclusion of community-based studies, clinical studies and epidemiological surveys.
3. Publication in peer-reviewed journals and availability in English.
4. Presentation of original data on ADHD prevalence rates, risk factors, or sex-specific analysis.
5. Publication between 2000 and May 2023 to capture recent developments in the field.

**Table 1:** Overview of literature search.

Database	Keywords	Inclusion criteria	Exclusion criteria
PubMed	ADHD, attention-deficit/hyperactivity disorder, prevalence, Africa, sub-Saharan Africa, North Africa, children, adolescents	Focus on ADHD prevalence among children and adolescents in African countries. 2. Inclusion of community-based studies, clinical studies, and epidemiological surveys. 3. Publication in peer-reviewed journals and availability in English. 4. Presentation of original data on ADHD prevalence rates, risk factors, or gender-specific analysis. 5. Publication between 2000 and May 2023.	Focused solely on adult populations or individuals outside the specified age range. 2. Commentaries or conference abstracts without original data. 3. Need more data or methodological limitations affecting the validity of results.
PsycINFO	ADHD, attention-deficit/hyperactivity disorder, prevalence, Africa, sub-Saharan Africa, North Africa, children, adolescents		
Google Scholar	ADHD, attention-deficit/hyperactivity disorder, prevalence, Africa, sub-Saharan Africa, North Africa, children, adolescents		

On the other hand, studies were excluded from the review if they:

1. Focused solely on adult populations or individuals outside the specified age range.
2. Were reviews, commentaries, or conference abstracts without original data.
3. Need more data or methodological limitations affecting the validity of results.

The data extracted from the selected studies were synthesised using a narrative synthesis approach to provide an overview of ADHD prevalence in Africa. This method summarised the findings and identified common themes and patterns across the studies. The synthesis focused on prevalence rates and sex-specific differences and identified risk factors for ADHD in the context of African communities.

By conducting this comprehensive review, we aim to shed light on the prevalence and risk factors associated with ADHD in Africa while also exploring the influence of sex and cultural factors on presenting and managing the disorder in African children and adolescents. The findings from this review will contribute to a better understanding of ADHD in the African context and potentially inform targeted interventions and strategies to address the disorder in the region.

### Prevalence and cultural perspectives of ADHD in Africa

In the context of Africa, with a total of 54 countries, our study’s findings highlight the heterogeneous nature of ADHD prevalence, which carries significant implications for public health initiatives and clinical practices in the region. The observed variation in prevalence rates across different regions and periods indicates that ADHD is not uniformly distributed throughout Africa but is influenced by a complex interplay of cultural factors, healthcare access and diagnostic practices.

Early studies conducted by Ashenafi *et al*. in Ethiopia and Kashala *et al*. in Kinshasa, the Democratic Republic of the Congo, reported discrepant prevalence rates of 1.49 and 6%, respectively^4,5^. These disparities exemplify the need for caution when generalising ADHD prevalence in Africa. Cultural norms surrounding child behaviour and attention and varying healthcare infrastructure and resources in different regions might impact how ADHD symptoms are perceived, recognised and diagnosed.

A sex-specific analysis of ADHD prevalence conducted by Kashala *et al*. revealed interesting patterns, with men exhibiting a higher prevalence of 7.1 compared to 4.9% in women^5^. This echoes global trends and emphasises the importance of investigating sex-specific risk factors and biological mechanisms that may contribute to this disparity. Societal expectations surrounding sex roles could also affect how ADHD symptoms are manifested and interpreted in males versus females.

Subsequent studies conducted in Nigeria and Egypt by Ofovwe *et al*. and Farahat *et al*. reported 8 and 6.9% prevalence rates^6,7^. These studies suggest a rising prevalence of ADHD in specific African countries, possibly indicating increased recognition of the disorder and improved access to healthcare services. However, further research is needed to understand the factors contributing to this trend and determine if it reflects a substantial rise in the disorder’s incidence or enhanced awareness and detection.

Recent studies in the last decade have added complexity to the ADHD prevalence landscape in Africa. Research by Bishry *et al*.^8^ on adolescent students reported a prevalence of 9.4%, suggesting a potential rise in ADHD prevalence among this age group. In contrast, studies in South Africa by Coetzee *et al*. and Hogan *et al*. reported significantly higher prevalence rates of 36 and 12.4%, respectively^9,10^. These figures warrant attention from policymakers and public health authorities to understand the underlying reasons for such high rates. Disparities in healthcare access, socioeconomic conditions and ADHD awareness may be contributing factors.

The systematic review and meta-analysis by Ayano *et al*.^11^ provided a pooled estimate of ADHD prevalence at 7.47% among children and adolescents in Africa. This analysis consolidates data from multiple studies, offering a more robust perspective on the regional disorder’s burden. However, the sex-specific analysis revealing a higher prevalence among males calls for further exploration of cultural, social and biological determinants contributing to this difference.

The consistent finding across the reviewed studies of males having a higher risk of developing ADHD than females, with a threefold increased likelihood, aligns with global trends. Understanding the sex-specific risk factors for ADHD in Africa is crucial for tailoring interventions and providing targeted support to those affected. Further research is needed to elucidate the underlying mechanisms contributing to this sex-based variation.

The findings regarding age as a crucial factor in ADHD prevalence underscore the significance of early intervention and support. The study by Wamulugwa *et al*.^12^ highlighted that children under 10 were four times more likely to have ADHD. This emphasises identifying and addressing ADHD symptoms during early childhood to minimise potential cognitive and psychosocial development impacts. Early detection and intervention programs can facilitate timely access to appropriate resources and therapeutic interventions, improving long-term outcomes for affected children.

Illiteracy among primary caregivers emerged as a notable risk factor for ADHD in African children, consistently supported by various studies^13,14^. Limited or no education among primary caregivers is associated with a higher risk of ADHD development. This finding highlights the importance of educational support and awareness programs targeting caregivers in vulnerable communities. Providing accessible and culturally sensitive education on ADHD symptoms, management strategies and available resources can empower caregivers to recognise early signs of ADHD and seek appropriate assistance for their children.

Family-related factors also play a significant role in ADHD cases, as highlighted by various researchers, including van Dyk *et al*., Lola *et al*., and Olashore *et al*.^15–17^. Factors such as living with a single parent, parental mental health disorders and nonmaternal child care influence ADHD prevalence. These findings emphasise the need for a holistic approach to understanding ADHD in Africa. Addressing family-related risk factors, promoting mental health support for parents and implementing family-based interventions can be crucial in mitigating the impact of ADHD on affected children and fostering a supportive environment for their well-being.

### Challenges with managing ADHD in Africa

Managing ADHD can be challenging in any part of the world, but specific difficulties are faced in Africa due to various factors, including limited resources and cultural perspectives Table 2.

**Table 2:** Challenges with managing ADHD in Africa and their implications.

Challenges	Description	Implications
Lack of awareness and stigma	Limited public awareness and understanding of ADHD in African communities. The stigma associated with the condition leads to misconceptions, discrimination, and social exclusion of individuals with ADHD.	Delayed identification and intervention for children with ADHD, resulting in missed early support and treatment opportunities.
	Reluctance of individuals and families to seek help for ADHD due to fear of judgment and societal disapproval.	
	Reduced acceptance and support for children with ADHD in educational settings and communities, potentially leading to academic and social difficulties.	
Limited access to mental health services	Inadequate accessibility of mental health services, particularly in rural and underserved areas. Lack of specialised professionals and facilities for diagnosing and treating ADHD.	Prolonged waiting times and delayed diagnosis for children with ADHD, leading to unaddressed challenges and potential worsening of symptoms.
	Disparities in ADHD care between urban and rural areas, with children in remote regions facing significant barriers to accessing essential services.	
Inadequate education system support	Education systems often lack the necessary accommodations and resources to support students with ADHD effectively.	Academic difficulties and underachievement for children with ADHD, hindering their educational prospects.
	Increased behavioural challenges in the classroom due to a lack of understanding and tailored interventions, affecting the overall learning environment for all students.	
	Greater risk of dropout and school disengagement among children with ADHD, further limiting their opportunities for personal and professional growth.	

#### Lack of awareness and stigma

African communities are confronted with a lack of awareness and understanding concerning ADHD^18^. This information gap leads to widespread misconceptions and a stigma surrounding the condition, making life challenging for individuals with ADHD^19^. Many individuals, including parents, educators and community members, remain unfamiliar with the neurobiological underpinnings of the disorder^18^. As a result, they often misinterpret the symptoms, viewing them as mere manifestations of misbehaviour, a lack of discipline, or deliberate defiance^20^. Such misunderstandings give rise to harsh judgments, criticism and, occasionally, punitive actions against children and adults with ADHD, exacerbating their difficulties and significantly impacting their overall well-being^21^. Consequently, affected individuals may internalise negative stereotypes about themselves, leading to feelings of shame and diminished self-esteem^22^. Consequently, they may avoid seeking assistance or treatment, fearing further marginalisation and censure^22^.

The consequences of this stigmatisation have far-reaching effects on individuals with ADHD and their families, creating formidable barriers to accessing the necessary support. Families often deny the existence of ADHD, hoping that the symptoms will fade over time or attributing the behaviours to other causes^23^. Seeking professional help may be perceived as a sign of weakness or failure, discouraging families from pursuing intervention^23^. Consequently, individuals with ADHD may not receive timely diagnosis and access to effective treatments, significantly impacting their educational and occupational prospects^22^.

The implications of this lack of awareness and stigma on a child’s education are equally profound. Educators and teachers, often uninformed about ADHD, may misinterpret the behaviours as deliberate disruptions, leading to disciplinary measures rather than providing appropriate support^24^. Consequently, the child’s academic struggles may intensify, impeding their educational progress^24^. Moreover, undiagnosed and untreated ADHD can cause difficulties in concentration, task completion and positive peer relationships, further compromising academic performance^24^.

The social consequences of stigma can be equally devastating. Individuals with ADHD may withdraw from social interactions to avoid judgment or ridicule, leading to feelings of isolation and loneliness^25^. Given that social support is crucial for mental well-being, the community’s lack of understanding and acceptance can contribute to heightened psychological distress for those grappling with ADHD^25^.

#### Limited access to mental health services

In several African countries, a significant impediment to the effective management of ADHD emerges from limited access to mental health services. Insufficient funding and inadequate attention to mental health contribute to the compromised state of mental health services in these regions, resulting in a lack of comprehensive care for those in need^26^. This scarcity of resources also extends to the availability of qualified professionals, particularly child psychologists and psychiatrists specialising in ADHD, thereby creating challenges in the early identification and intervention of children with the condition^27^. The dearth of trained mental health professionals with expertise in ADHD poses considerable obstacles for families seeking assistance for their children. Consequently, numerous children with ADHD may remain undiagnosed or receive inaccurate diagnoses, prolonging their struggles and impeding their developmental progress^11^.

Without timely intervention and appropriate treatment, children with ADHD confront difficulties in academic performance, behavioural challenges and social interactions^28^. Early identification and access to evidence-based interventions, such as behavioural therapy and medication, are essential for effectively managing ADHD and enabling these children to realise their full potential^29^. Moreover, the repercussions of limited access to mental health services extend beyond childhood and can have enduring effects on individuals’ lives. The unmet mental health needs of children with ADHD may persist into adulthood, significantly influencing their personal and professional trajectories^29^.

#### Education system challenges

In certain African countries, the education system encounters significant challenges that impede its ability to support and accommodate children with ADHD^30^ adequately. A prominent obstacle is the prevalence of large class sizes in many schools^30^. The overcrowded classrooms hinder teachers from providing personalised attention to each student, leaving those with ADHD without the requisite support^30^. Consequently, these children may need help to keep up with the fast-paced instruction and become disengaged from the learning process.

Moreover, limited resources present another barrier to effectively addressing the needs of students with ADHD. The demand for educational materials, specialised tools and support staff makes it challenging for teachers to implement effective strategies for these students^31^. Consequently, the academic performance and overall well-being of students with ADHD may suffer.

Compounding the issue is the inadequate training of teachers concerning neurodevelopmental disorders like ADHD^32^. The lack of awareness and understanding can lead to misconceptions about the condition, resulting in students with ADHD being misunderstood and their behaviours inaccurately attributed to a lack of effort or discipline. Regrettably, in some instances, disciplinary measures may be taken against these students, further exacerbating their struggles and hindering their access to appropriate support.

Due to these challenges, students with ADHD risk being overlooked within the education system^32^. Their specific needs may not be recognised, and they may not receive the necessary accommodations or interventions. Consequently, these children may experience discouragement and isolation, sensing that they are left behind and unsupported in their academic journey.

#### Economic constraints

In Africa, the management of ADHD is often complicated by economic challenges. The treatment of ADHD typically involves a range of therapies, medications and interventions, many of which can be financially demanding^33^. However, in regions where a significant portion of the population lives in poverty, such as African low-income communities, families may encounter difficulties affording the necessary treatments or accessing healthcare facilities in urban areas^34^.

The cost of managing ADHD can substantially burden families already struggling to meet their basic needs^35^. Behavioural therapies, counselling sessions and specialised educational interventions play vital roles in ADHD management, but they often come with significant expenses^36^. Additionally, the medications prescribed to manage ADHD symptoms may be prohibitively expensive, limiting accessibility for many financially stretched families^37^.

Families residing in remote or rural areas face additional challenges, as access to specialised healthcare facilities is often limited^37^. The impracticality and high cost of travelling to urban centres for ADHD treatment, particularly in regions with inadequate transportation infrastructure, pose significant barriers. Consequently, many families may find themselves compelled to forgo seeking professional help for their children with ADHD, leaving these individuals without the necessary support to manage their condition effectively.

The economic constraints experienced by families in low-income communities can have far-reaching consequences for children with ADHD. The lack of access to appropriate treatments and interventions may result in difficulties with academic performance, social interactions and overall well-being. Furthermore, their educational and vocational opportunities may be curtailed, leading to long-term implications for their prospects and economic mobility.

#### Cultural beliefs and practices

Cultural beliefs and practices hold significant sway over the perception and treatment of ADHD within Africa^38^. In certain cultures, behaviours characteristic of ADHD, such as hyperactivity, impulsivity and inattentiveness, may be attributed to spiritual possession or witchcraft rather than being recognised as neurodevelopmental symptoms^39^. This misunderstanding can stigmatise and discriminate against individuals with ADHD, as they may be unfairly regarded as ʻpossessedʼ or ʻcursedʼ.

The misattribution of ADHD behaviours to spiritual causes can have profound and detrimental consequences. People with ADHD may face ridicule, ostracism, or even be subjected to harmful traditional treatments, such as exorcism rituals, instead of receiving appropriate medical care^40^. The reluctance to seek professional help due to cultural beliefs can lead to delays in diagnosis and intervention, exacerbating the challenges faced by individuals with ADHD and impeding their ability to lead fulfilling lives^40^.

Furthermore, the stigma associated with mental health conditions like ADHD can extend beyond the affected individuals to their families^23^. Parents may experience feelings of shame or fear of social repercussions, leading them to conceal their child’s condition and avoid seeking support. This culture of secrecy can further isolate the child and hinder their access to necessary resources and understanding from their community^23^.

### Policy recommendations and future directions for ADHD in Africa

Over the years, efforts to address ADHD in Africa have gained momentum, with various stakeholders working to enhance awareness, diagnosis, treatment and support for individuals impacted by the disorder. Collaboration with international organisations, such as the WHO and UNICEF, has further accelerated progress in the field of ADHD in Africa. By forging partnerships with global entities, African countries gain access to valuable resources, knowledge and best practices that can be adapted to suit local contexts. This collaborative approach fosters a dynamic exchange of ideas and expertise, contributing to a comprehensive and sustainable approach to ADHD management. However, prioritising public awareness campaigns and educational programs is crucial to address the challenges surrounding ADHD in Africa Table 3. Public awareness campaigns are essential for disseminating accurate information about ADHD. Governments and health organisations can utilise various media platforms such as television, radio, social media and community outreach programs to reach a wide audience. These campaigns should focus on explaining the symptoms, causes and impact of ADHD on individuals and their families. These initiatives should target various segments of society, including the general public, educators, healthcare professionals, religious leaders and traditional healers. Additionally, providing information on evidence-based treatment options can empower affected individuals and their caregivers to seek appropriate help.

**Table 3:** Proposed interventions for equitable ADHD care in Africa and likely outcomes.

Interventions	Description	Likely outcomes
Public awareness campaigns	Conduct comprehensive public awareness campaigns to disseminate accurate information about ADHD, its prevalence, symptoms, and available treatments.	Increased awareness and understanding of ADHD in the general population. Reduced stigma and misconceptions surrounding the condition. Timely identification and referrals of children with ADHD for appropriate evaluation and support. Improved acceptance and support for individuals with ADHD in communities.
Training for healthcare professionals	Equip healthcare professionals, including primary care providers and specialists, with the latest research and evidence-based practices.	Enhanced competence of healthcare professionals in diagnosing and managing ADHD. Improved accuracy in identifying ADHD symptoms and appropriate referral to specialised services. Early intervention and tailored treatment plans for children with ADHD. Increased collaboration between healthcare professionals and other stakeholders in providing comprehensive care for children with ADHD.
Integration of mental health services	Integrate mental health services into primary healthcare systems to improve access to ADHD diagnosis and treatment. Train primary care providers to recognise ADHD symptoms and offer initial support and referrals for further evaluation and intervention.	Increased availability and accessibility of ADHD diagnosis and treatment in primary healthcare settings. Reduced burden on specialised mental health facilities, providing more focused support to severe cases. Enhanced coordination and communication between primary care providers and mental health specialists for comprehensive ADHD care.
Addressing affordability and accessibility	Implement policy measures to make essential ADHD treatments and medications more affordable.	The reduced financial burden on families seeking ADHD treatment. Improved affordability and access to medications and therapies for children with ADHD. Increased treatment adherence and compliance due to reduced financial constraints. Expanded access to necessary interventions, especially in underserved communities.
Telemedicine and digital health platforms	Utilise telemedicine and digital health platforms to improve the accessibility of ADHD treatment, especially in remote or underserved areas.	Improved access to expert advice and consultation for individuals with ADHD, regardless of geographic location. Enhanced convenience and flexibility in accessing ADHD support and resources. Reduced barriers to care, especially for families in remote or underserved areas. Efficient and effective delivery of information and treatment recommendations through digital platforms.
Stakeholder collaboration	Establish public-private partnerships between governments, NGOs, and pharmaceutical companies to reduce the costs of medications and therapies. Collaborate to develop innovative solutions and increase the availability of resources for managing ADHD.	Increased availability of affordable medications and therapies for ADHD management. Greater investment in research and resources for improving ADHD care. Innovative solutions for addressing unique challenges in African communities. Enhanced collaboration and collective

Educators play a vital role in the lives of children with ADHD as they spend a significant amount of time with them in schools^41^. It is crucial to include educators in awareness initiatives by organising workshops, training programs and seminars to equip them with the knowledge and skills to recognise ADHD symptoms in their students. This enables early identification and timely interventions and support in the classroom setting. Similarly, healthcare professionals, including primary care providers and specialists, diagnose and treat ADHD^42^. Ensuring they are well informed about the latest research, diagnostic criteria and evidence-based treatments is crucial. Continuing medical education programs focused on ADHD can be organised to keep healthcare providers up-to-date with the best practices for managing the condition effectively. Moreover, religious leaders and traditional healers are highly respected figures in African communities^43^, so their influence can be significant. By involving them in awareness campaigns, they can become advocates for understanding and supporting individuals with ADHD. Providing them with accurate information about the neurodevelopmental nature of ADHD can help dispel misconceptions and reduce the stigma surrounding the condition. In turn, they can be crucial in encouraging community acceptance and support for those affected.

In addition to raising awareness about ADHD, strengthening mental health services in Africa is paramount to addressing the challenges associated with ADHD management effectively. Adequate funding should be allocated to mental health programs and facilities, with special attention given to rural and underserved areas. Investing in these regions ensures that individuals with ADHD can access the necessary support and treatment options regardless of location. In addition, expanding the availability of trained mental health professionals, such as child psychologists and child psychiatrists, is critical to improving the diagnosis and treatment of ADHD. These specialists possess the expertise to accurately diagnose ADHD and develop tailored treatment plans based on each child’s unique needs. Ensuring sufficient qualified professionals can reduce waiting times for assessments and interventions, allowing children with ADHD to receive timely and appropriate care.

Integrating mental health services into primary healthcare systems is another essential step. Since primary care providers are often the first point of contact for families seeking medical assistance, training them to recognise ADHD symptoms is vital. Equipping them with knowledge and resources to offer initial support and referrals can lead to early identification and intervention for children with ADHD. This integrated approach can bring mental health services closer to the communities, making it more convenient for families to seek help.

Addressing the affordability and accessibility of ADHD treatment is critical to ensuring that individuals with the condition receive the support they need, regardless of their economic circumstances. In Africa, where many families face diverse economic challenges and limited access to healthcare resources, the cost of managing ADHD can pose a significant burden. Policy measures must be implemented to make necessary treatments and interventions more affordable and accessible. One key policy intervention is the subsidisation of medications and therapies for ADHD. Medications like stimulants, often prescribed to manage ADHD symptoms, can be costly for many families. By implementing subsidies or price controls on these medications, governments can reduce the financial strain on families and increase access to essential treatment. Pharmaceutical companies can also play a role in ensuring affordability by working with governments to offer discounted or lower-cost medications for ADHD management. In addition to medication subsidies, therapies and interventions for ADHD should be made more affordable. Behavioural therapies, counselling and educational support are crucial components of comprehensive ADHD management, but their costs can be prohibitive for many families. Governments can partner with nongovernmental organisations (NGOs) to establish subsidised or free therapy programs for children with ADHD. These programs can be implemented in schools or community centres, making them easily accessible to affected individuals and their families.

Financial assistance programs can be instrumental in supporting families with limited resources. Governments and NGOs can create targeted financial aid programs to help families cover the costs of ADHD treatment. These programs can be means-tested to ensure that the families most in need receive the support they require. Such initiatives can significantly reduce the financial burden on families, encouraging them to seek timely and appropriate treatment for their children.

Furthermore, telemedicine and digital health platforms can play a role in improving the accessibility of ADHD treatment, especially in remote or underserved areas. Telemedicine enables healthcare professionals to provide virtual consultations, allowing individuals with ADHD to access expert advice and support regardless of their geographic location.

One of the strengths of this study lies in its comprehensive and diligent review of the existing literature on ADHD in Africa. By critically analysing available studies, the research provides valuable insights into the unique challenges faced in delivering equitable care to children with ADHD in the region. This inclusive examination allows a deeper understanding of the multifaceted factors influencing ADHD care, paving the way for informed and targeted interventions. Despite the study’s strengths, it is important to acknowledge the existing limitations, primarily stemming from the current state of data availability. The scarcity of studies on ADHD in Africa poses challenges in making conclusive comparisons across different countries. Nevertheless, the study makes the most of the available data and presents a comprehensive analysis within this context, providing valuable insights that can inform future research and policy development.

## Conclusion

Managing ADHD in Africa is a complex challenge influenced by social, economic and cultural factors. Limited awareness and stigma hinder timely support for affected individuals. Access to mental health services in rural areas delays ADHD diagnosis and treatment. The education system’s inability to accommodate neurodevelopmental disorders worsens students’ ADHD difficulties. Economic constraints in African communities make essential ADHD treatments unaffordable. Cultural beliefs lead to misunderstandings and inappropriate treatment. To address these challenges, prioritise public awareness campaigns and education to dispel misconceptions and reduce stigma. Strengthen mental health services and train professionals in ADHD diagnosis and treatment for early intervention. Improve affordability and accessibility through subsidies, financial aid and stakeholder collaboration. Integrate appropriate traditional healing practices to foster cultural acceptance. These measures require concerted efforts from governments, healthcare professionals, educators, communities and stakeholders. Further research, resource allocation and advocacy are vital to support children with ADHD and unlock their potential for a brighter future.

## Ethical approval

Ethical approval is not applicable for this review.

## Consent

Informed consent is not applicable for this review.

## Sources of funding

No funding was received for this review.

## Author contribution

N.A.: conceptualization. All authors contributed in writing of first and final drafts.

## Conflicts of interest disclosure

All authors declare no conflicts of interest.

## Research registration unique identifying number (UIN)

1. Name of the registry: not applicable.
2. Unique identifying number or registration ID: not applicable.
3. Hyperlink to your specific registration (must be publicly accessible and will be checked): not applicable.

## Guarantor

Nicholas Aderinto.

## Data availability statement

No new dataset were generated for this review.

## Provenance and peer review

Not commissioned, externally peer-reviewed.
